# Analyzing and validating the prognostic value and immune microenvironment of clear cell renal cell carcinoma

**DOI:** 10.1080/19768354.2022.2056635

**Published:** 2022-03-29

**Authors:** Jingwei Ke, Jie Chen, Xin Liu

**Affiliations:** a Department of urology, The Affiliated Traditional Chinese Medicine Hospital of Southwest Medical University, Luzhou, People’s Republic of China; b Graduate School of Southwest Medical University, Luzhou, People’s Republic of China

**Keywords:** Clear cell renal cell carcinoma, microenvironment, prognosis, nomogram, bioinformatics

## Abstract

Tumor immune microenvironment (TIME) plays an important role in tumor diagnosis, prevention, treatment and prognosis. However, the correlation and potential mechanism between clear cell renal cell carcinoma **(**ccRCC) and its TIME are not clear. Therefore, we aimed to identify potential prognostic biomarkers related to TIME of ccRCC. Unsupervised consensus clustering analysis was performed to divide patients into different immune subgroups according to their single-sample gene set enrichment analysis (ssGSEA) scores. Then, we validated the differences in immune cell infiltration, prognosis, clinical characteristics and expression levels of HLA and immune checkpoint genes between different immune subgroups. Weighted gene coexpression network analysis (WGCNA) was used to identify the significant modules and hub genes that were related to the immune subgroups. A nomogram was established to predict the overall survival (OS) outcomes after independent prognostic factors were identified by least absolute shrinkage and selection operator (LASSO) regression and multivariate Cox regression analyses. Five clusters (immune subgroups) were identified. There was no significant difference in age, sex or N stage. And there were significant differences in race, T stage, M stage, grade, prognosis and tumor microenvironment. WGCNA revealed that the red module has an important relationship with TIME, and obtained 14 hub genes. In addition, the nomogram containing *LAG3* and *GZMK* accurately predicted OS outcomes of ccRCC patients. *LAG3* and *GZMK* have a certain correlation with the prognosis of ccRCC patients, and play an important role in the TIME. These two hub genes deserve further study as biomarkers of the TIME.

## Introduction

Renal parenchymal carcinoma is adenocarcinoma derived from renal tubular epithelial cells; 85% of these tumors are clear cell carcinomas, while others are granular cell carcinomas and mixed cell carcinomas. Hemorrhage, necrosis, cystic change and calcification are common in cancer (Bray et al. 2018). Clear cell renal cell carcinoma (ccRCC) has the highest mortality rate among urinary system cancers. Its incidence rate and mortality rate account for 2% of all tumors. Approximately 100 thousand patients worldwide die of this disease (Torre et al. 2016). China's incidence rate of renal cancer is approximately 3%, and the incidence rate of renal cancer in cities (4.73/10^5^) is significantly higher than that in rural areas (1.89/10^5^) (Pang et al. 2016). Approximately 30% of ccRCC is advanced when diagnosed, and approximately 10%-20% of early ccRCC will recur and metastasize after treatment. The 5-year survival rate is approximately 11.7% (La Rochelle et al. 2010).

Compared with other tumors, ccRCC is less sensitive to chemotherapy and radiotherapy, and surgery is still the main treatment for nonadvanced ccRCC patients. Many years ago, the drug treatment of advanced ccRCC was limited to interleukin-2 and interferon alpha. However, due to its serious adverse reactions and extremely low efficiency, its clinical application is not widely used (Escudier et al. 2007). The development and application of immune checkpoint inhibitors (ICIs) have improved the prognosis of patients with RCC. The CTLA-4 pathway plays an important role in the early stage of immune system activation, and the PD-1 / PD-L1 pathway plays an important role in immune system tumor microenvironment and is related to tumor immune escape mechanism (Zhou et al. 2020). The first-line treatment of advanced ccRCC with ravulizumab combined with ipilimumab reported in 2018 confirmed the first-line treatment status of this regimen in advanced renal clear cell carcinoma. A total of 1096 patients with advanced renal clear cell carcinoma were enrolled in this study. They were randomly divided into two groups: the ravulizumab combined with ipilimumab group and the sunitinib group. The objective remission rate of the combined group was significantly higher than that of the sunitinib group (42% vs. 27%, *P* < 0.001), and the complete remission rate of the combined group was also higher than that of the sunitinib group (9% vs. 1%). After a median follow-up of 25.2 months, the survival rates of the combined group and sunitinib group were 75% and 60%, respectively (Motzer et al. 2018).

Due to the success of immunotherapy, countless patients have achieved remarkable results with its intervention. However, some patients did not respond to immunotherapy. The complexity and diversity of the tumor immune microenvironment (TIME) may determine its important impact on immunotherapy (Yang and Zhang 2020; Shang et al. 2018). Immune cells and stromal cells are two important components of the extracellular matrix in the microenvironment. They have a wide range of interactions with tumor cells, including early tumor recruitment and the activation of stromal cells to form primitive precancerous stroma. These cells can change the cancer cell phenotype to a malignant phenotype. They also establish complex cell–cell interaction networks that help to improve and maintain the immunosuppressive microenvironment, promote immune escape, and ultimately promote the development of cancer. Therefore, the TIME is a multidirectional, dynamic and complex interaction network among immune cells, stromal cells and tumor cells (Hanahan and Coussens 2012). Therefore, in this study, we aimed to identify possible biomarkers for ccRCC through the tumor immune microenvironment.

## Materials and methods

### Data collection and processing

In RNA-Seq analysis, normalizing the number of read counts of genes or transcripts is an extremely important step, because the number of read counts falling in a gene region depends on gene length and sequencing depth. When we analyze gene differential expression, we often compare the expression of different genes in multiple samples. If we do not standardize the data, the comparison results are meaningless. Therefore, the two key factors that we need to standardize are gene length and sequencing depth. Fragments per kilobase of transcript per million mapped reads (FPKM) is often used as the standardized value. Level 3 HTSeq-FPKM RNA-seq data of ccRCC patients were downloaded from the UCSC Xena database (https://xenabrowser.net/datapages/) (Goldman et al. 2015), and clinical information for each patient was obtained. Samples with any incomplete information were excluded, and patients who survived for less than 1 day were also excluded. We definded overall survival (OS) as the period from the diagnosis to death for any reason and progression-free survival (PFS) as the period from the diagnosis to death or progression of the tumor.

### Evaluation of the immune cell infiltration level

Single-sample gene set enrichment analysis (ssGSEA) (Barbie et al. 2009) was performed to obtain the 29 immune signatures, including immune cells or immune function, of each sample, and the activities of the immune pathways were assessed using the GSVA package (Hänzelmann et al. 2013). In the tumor microenvironment, immune cells and stromal cells are two main types of nontumor components and have been proposed for the diagnostic and prognostic evaluations of tumors. The ESTIMATE algorithm (Wilkerson and Hayes 2010) was used to evaluate the immune cell infiltration level (immune score), stromal content (stromal score) and comprehensive environmental score (ESTIMATE score).

### Identification of molecular subtypes

Consistent clustering is a method used to provide quantitative evidence for determining the number and members of possible clusters in a dataset (e.g. a microarray gene expression dataset). The consistency clustering method involves subsampling from a group of items and determining clusters with a specific cluster count (k). Then, for the consensus value, two items with the same clustering in the same subsample are calculated and stored in the symmetric consistent matrix of each k value. The ConsensusClusterPlus package (Yoshihara et al. 2013) was used to perform consistent clustering analysis of each ssGSEA score of each ccRCC sample.

### Weighted Gene Coexpression Network Analysis (WGCNA)

The WGCNA package (Langfelder and Horvath 2008) was used to perform this analysis. We first selected the genes that varied greatly in different samples (i.e. genes whose variance was greater than all variance quartiles). The goodsamplesgenes function was used to evaluate whether the matrix data were qualified, that is, whether there was a missing value. If so, the gene was excluded. Then, the samples were clustered, and outliers were removed. After removing the outliers, we reconstructed the sample clustering tree and selected an appropriate soft threshold (*β*) to ensure scale-free distribution; that is, a small number of genes showed an absolute advantage (high expression), and most showed a disadvantage (low expression). By means of the power index, a certain power index was used to determine the correlation coefficient between genes, and a suitable soft threshold (*β*) was determined. According to the *β* value, the proximity matrix and topological matrix were obtained, and the genes were clustered by dissimilarity between genes. Then, the tree was cut into different modules by the dynamic cutting method (the minimum number of genes in the module was 50). Combining the correlation between each module and sample character, we analyzed the relationship between the module and phenotype. The module with the highest correlation coefficient with clinical characteristics was considered an important module. In addition, genetic significance (GS) and module membership (MM) were calculated. GS was defined as the level of correlation between gene expression and clinical characteristics. MM was defined as the correlation between the module's own genes and gene expression profiles. For the important modules, the inclusion criteria for the hub genes were as follows: GS >0.4 and MM > 0.7.

### Functional enrichment analysis

For important modules and hub genes, we analyzed their biological functions and possible pathways through Gene Ontology (GO) database and Kyoto Encyclopedia of Genes and Genomes (KEGG) analyses. The above analysis is performed by the clusterProfiler (Yu et al. 2012), org.Hs.eg.db and enrichplot packages. Fisher exact test and Benjamin Hochberg (B-H) multiple test correction method were used to correct the occurrence of false positive. The adjustied *P* < 0.05 was taken as the cut-off standard.

### Construction and Validation of the Nomogram

To further verify the impact of the hub genes on the prognosis of patients, we combined the clinical information and prognostic information to construct the relevant nomogram. Least absolute shrinkage and selection operator (LASSO) regression analysis was first used to filter variables and prevent overfitting using the glmnet package (Engebretsen and Bohlin 2019). After the influencing factors were screened by LASSO regression, multivariate Cox regression analysis was used to identify independent prognostic factors using the survival package (Rizvi et al. 2019). The nomogram was used to show the predictive results of these independent prognostic factors for the 1-, 3-, and 5-year OS rates by multivariate Cox regression analysis using the rms package (Huang et al. 2019). Calibration curves were generated to test the predictive power of the nomogram. In addition, a time-dependent receiver operating characteristic (ROC) curve was used to judge the predictive power of the nomogram.

### Statistical analysis

All the above calculations were carried out with R software (version 4.0.2, 64 bit). Corrected *P* < 0.05 was considered statistically significant. When comparing whether there was a significant difference between the two groups of data, we used the rank-sum test. To determine whether there was a significant difference between multiple groups of data, we used the Kruskal–Wallis test. The Kaplan-Meier method was used to draw the patient survival curves, and the log-rank test was used to compare whether there was any difference in prognosis between the groups.

## Results

### Immune microenvironment landscape of ccRCC

After using the ssGSEA method to measure the immune function of The Cancer Genome Atlas (TCGA) ccRCC transcriptome, we obtained the corresponding ssGSEA score of each sample according to the 29 immune-related pathways, and immune cell infusion was used to evaluate the immune function of ccRCC, as shown in Supplementary Table 1. R code is available in Supplementary Data Sheet S1.

### Consensus clustering according to molecular aubtypes

**Figure 1.**
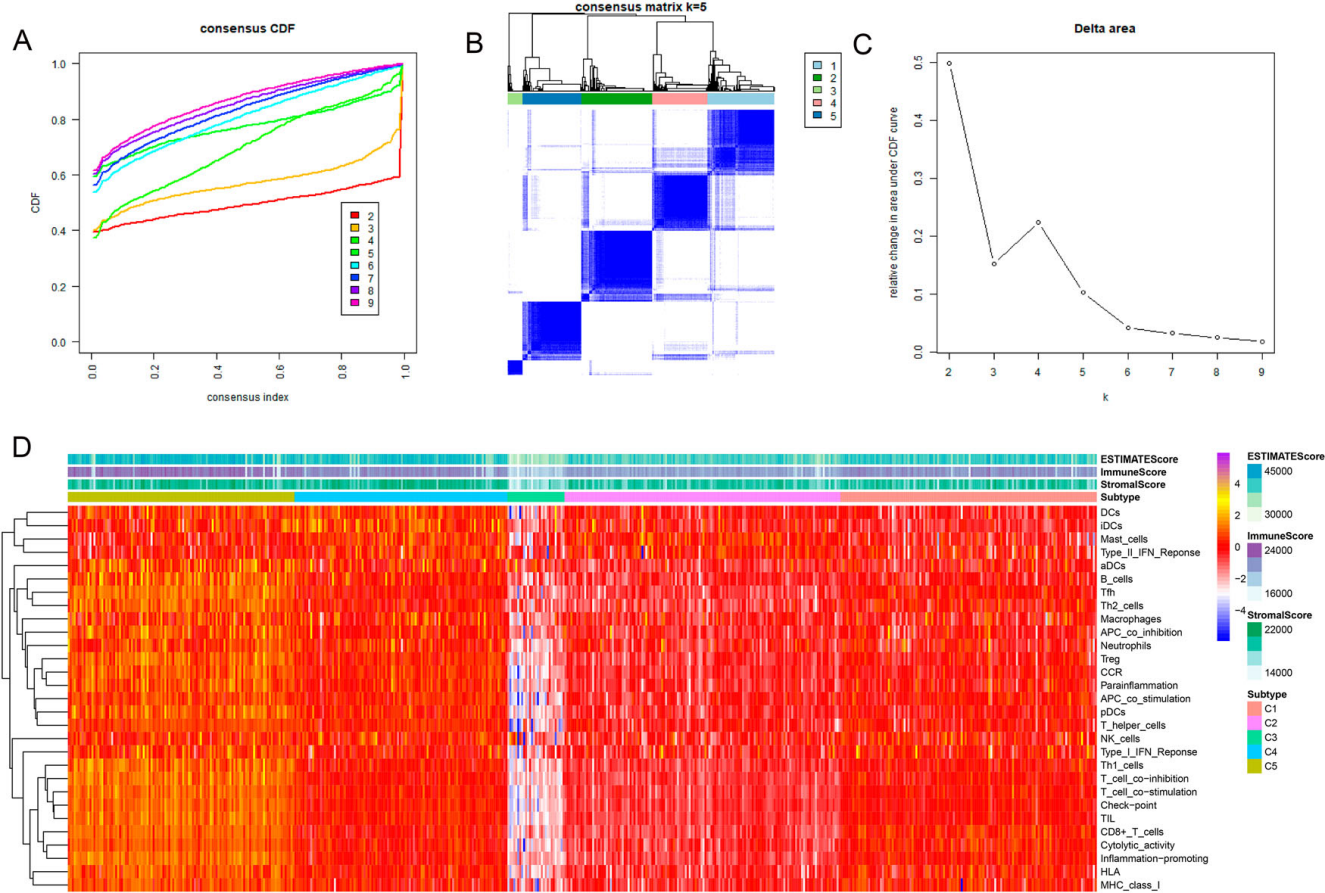
Consensus clustering results based on the ssGSEA scores of ccRCC patients. (**A**) Empirical cumulative distribution function (CDF) plot displaying consensus distributions for each k value. (**B)**. Consensus matrix heat map depicting consensus values on a white to blue color scale of each cluster. (**C)**. Delta area plot reflecting the relative changes in the area under the CDF curve. Usually, we choose the K value with a small gradient of CDF decline or the point where the delta area starts to be gentle as the best K value. In the follow-up, we need to combine the clinical data of the sample to determine whether these groups have practical significance. (**D**). Heat map displaying the profiles of the 5 clusters and corresponding stromal scores, immune scores and ESTIMATE scores.

The results of consensus clustering of the ssGSEA scores were visualized using an empirical cumulative distribution function (CDF) plot and a delta area plot (Figure 1A and C). K = 5 was regarded as the best value to cut these clusters, and the consensus matrix heat map is shown in Figure 1B. Therefore, the total ccRCC dataset was divided into 5 clusters: C1, C2, C3, C4, and C5. By using the ESTIMATE algorithm, we obtained three scores: the immune score, stromal score and ESTIMATE score (shown in Supplementary Table 2). A heatmap of the 5 clusters with their ssGSEA and ESTIMATE scores is shown in Figure 1D. Cluster C3 showed obvious differences compared with the other clusters.

**Figure 2.**
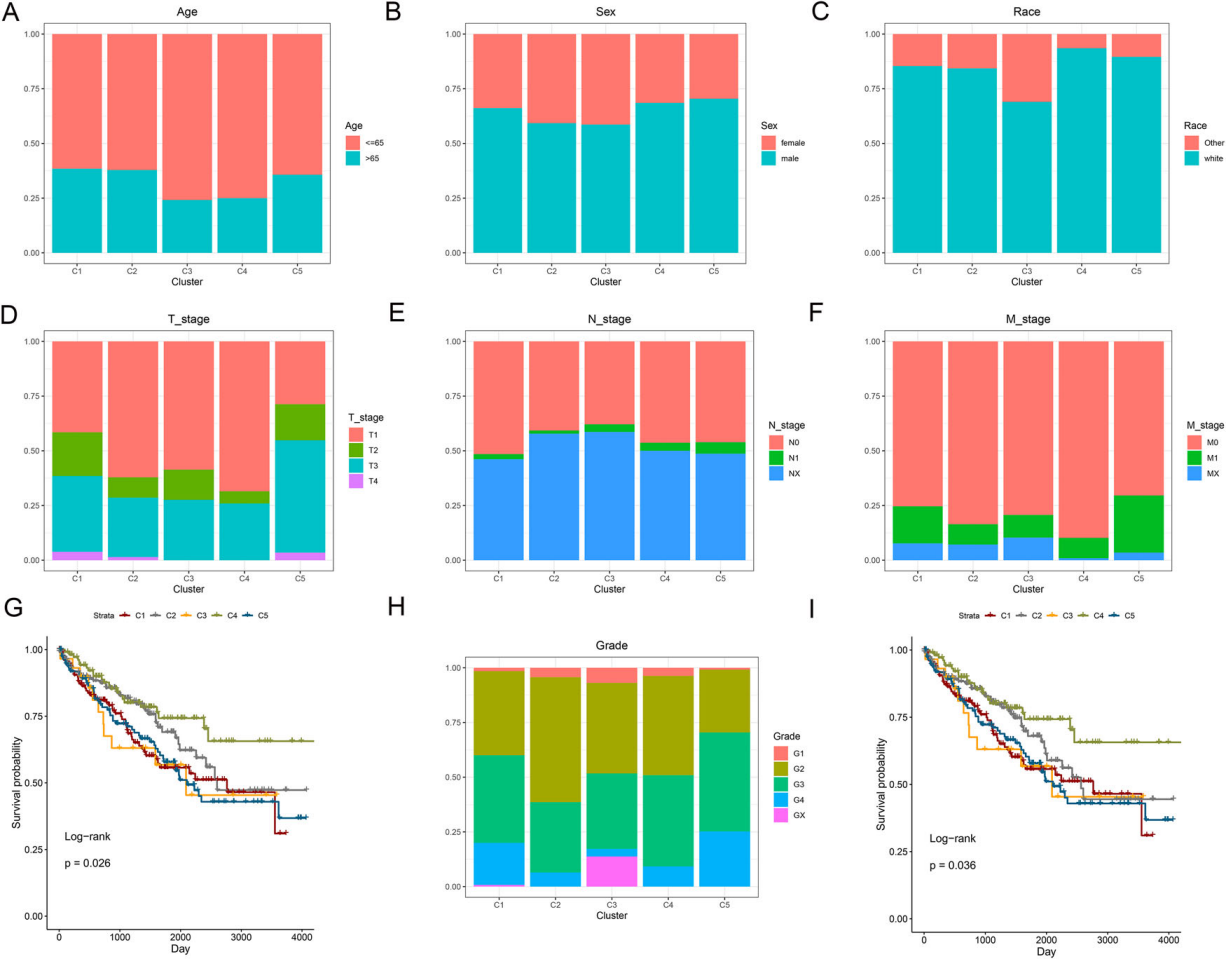
Distribution proportions based on (**A**) age, (**B**) sex, (**C**) race, (**D**) T stage, (**E**) N stage, (**F**) M stage, and (**H**) grade in each cluster. Survival curves comparing different clusters for OS (**G**) and PFS (**I**).

**Figure 3.**
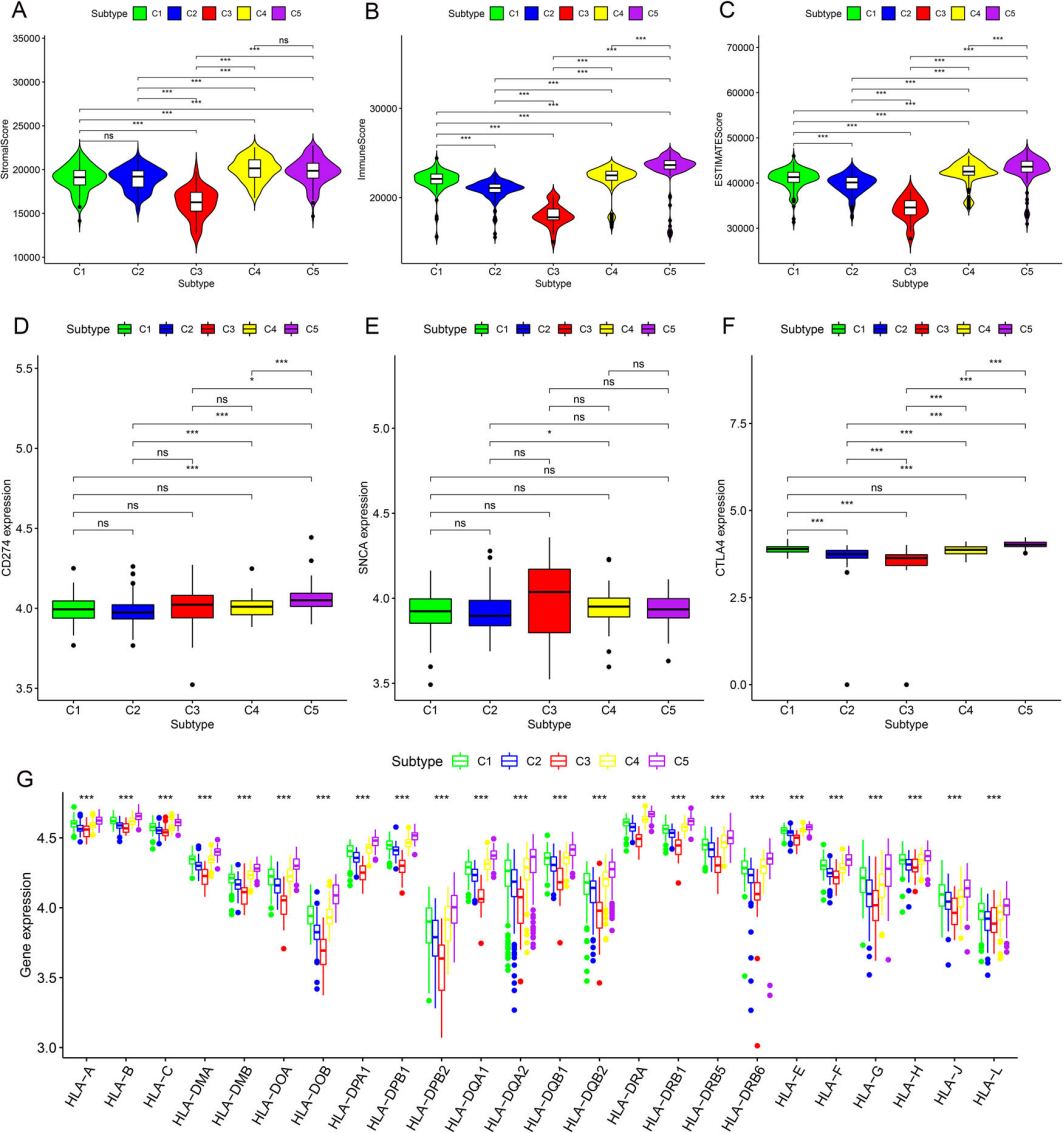
The difference of stromal scores (A), immune scores (B) and ESTIMATE scores (C). The difference of 3 immunosuppressive point. (A). CD274 (B). SNCA (C) CTLA4. The difference of HLA family for 5 clusters.

For different clusters, there was no statistical difference in age (Figure 2A, *P* = 0.1185), sex (Figure 2B, *P* = 0.3226) or N stage (Figure 2E, *P* = 0.3545). However, there were significant differences in race (Figure 2C, *P* = 0.0072), T stage (Figure 2D, *P*<0.0001), M stage (Figure 2F, *P* = 0.0052) and grade (Figure 2H, *P* = 0.0072). Regarding the prognosis of patients, there were significant differences between the five clusters. Cluster 4 had the best prognosis for both OS (Figure 2G) and PFS (Figure 2I). Moreover, the tumor microenvironment using the ESTIMATE algorithm was statistically significant between the five clusters (Figure 3A–C). However, concerning the stromal score, there was no significant difference between the C4 and C5 clusters (Figure 3A). For the three ICIs and PD1 and PDL1, only some clusters showed significant differences (Figure 3D and E). Regarding CTLA4, except for the expression of CTLA4 between cluster 1 and cluster 4, there were significant differences in the other clusters (Figure 3F). Notably, almost all HLA genes showed significant differences between the five clusters (Figure 3G).

### WGCNA

**Figure 4.**
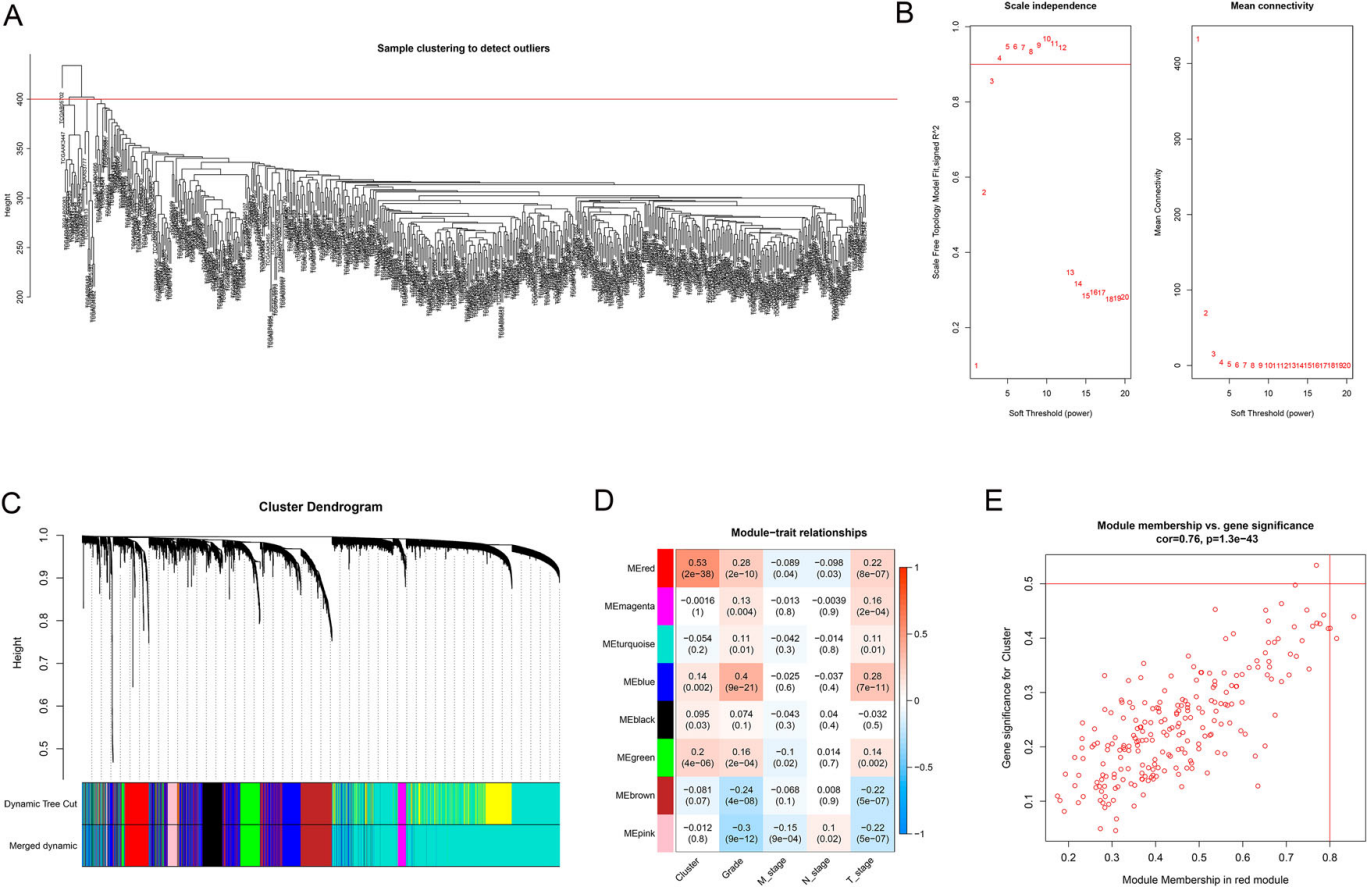
WGCNA results. (**A**) Cluster analysis of ccRCC patients and samples with a height above 400 are excluded as outliers. (**B**) Competition selection of the best soft threshold (*β*). (**C**) Hierarchical clustering tree shows each module. Each color of the tree represents a module. Gray modules are represented as genes that are not classified into any modules. (**D**) Correlation between the modules and clinical characteristics, including the different clusters. (**E**) Scatter diagram for MM vs. GS for the different clusters in the red module.

**Figure 5.**
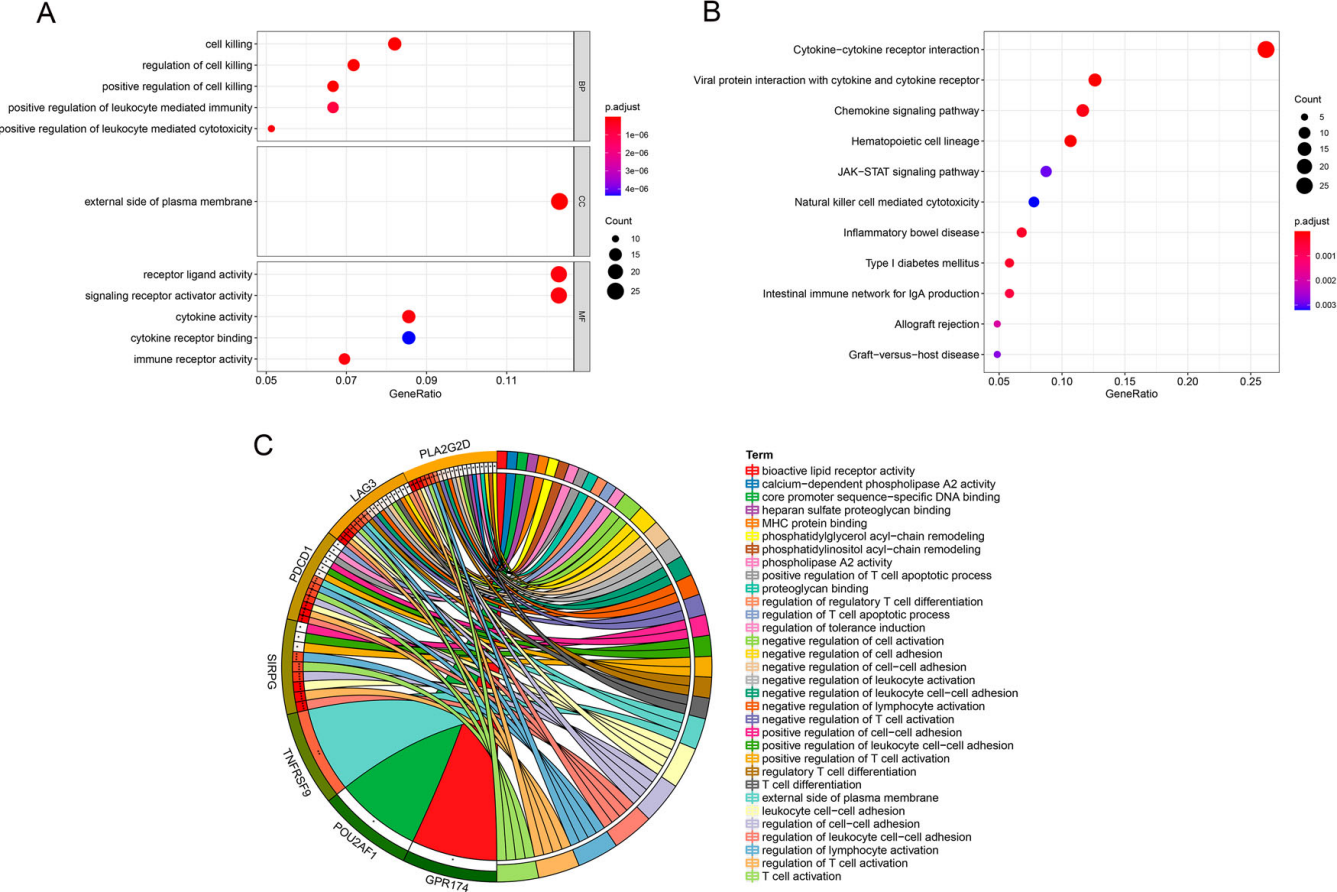
Enrichment analyses. (**A**) GO analysis of the red module. (**B**) KEGG pathway analysis of the red module. (**C**) Enrichment analyses of hub genes.

A total of 4555 genes and 526 samples with great variation in different samples were subjected to WGCNA. We first made it clear that all samples had no missing values, and sample cluster analysis suggested that an outlier was excluded.(Figure 4A). *β* = 3 was used to establish a proximity matrix to make our gene distribution conform to the scale-free network (Figure 4B). After clustering genes and using the dynamic cutting method to cut the tree into different modules and merge similar modules, we obtained a total of 8 modules (Figure 4C). Among them, the red module was positively correlated with immune cluster classification (*R*^2 ^= 0.53, *P* = 2e-38) (Figure 5D). The red module showed the highest GS and MM based on an intramodular analysis (Figure 5E) and was regarded as a significant module. Based on the cutoff value for hub genes (MM > 0.7 and cor GS > 0.4), we ultimately obtained 14 hub genes: *CD79A*, *FCRLA*, *GPR174*, *GZMK*, *JCHAIN*, *LAG3*, *MZB1*, *PDCD1*, *PLA2G2D* and *POU2AF1* (supplementary table 3).

### Functional enrichment analysis

In the GO analysis, the red modules were mostly enriched in cell killing, the external side of the plasma membrane and receptor ligand activity (Figure 5A). In the KEGG analysis, the red modules were mostly enriched in cytokine−cytokine receptor interactions (Figure 5B). The main enriched functions and pathways of the 14 hub genes were related to immunity. Examples included the regulation of regulatory T cell differentiation, the regulation of the T cell apoptotic process and the negative regulation of T cell activation (Figure 5C).

### Identification of the prognostic factors of nomogram

**Figure 6.**
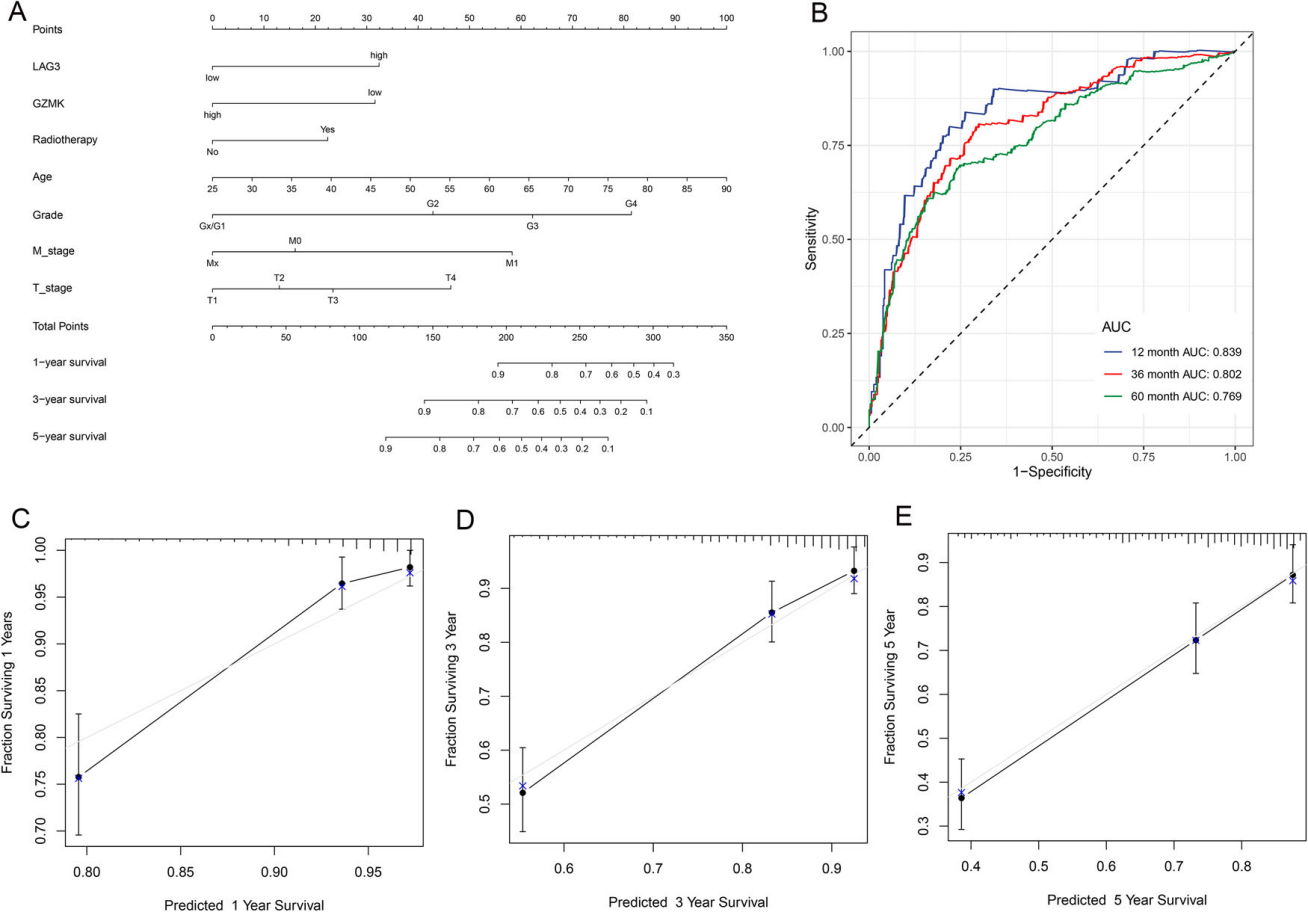
(**A**) The nomogram used to predict OS for ccRCC patients. (**B**)Time-dependent ROC curve for 1-, 3-, and 5-year OS proved the great prediction ability. Calibration curves for predicting 1 – (**C**), 3 – (**D**), and 5-year (**E**) OS probability.

Eleven prognostic factors, including three hub genes, *MZB1*, *GZMK* and *LAG3* were identified by LASSO regression analysis (supplementary figure 1). Multivariate Cox regression analysis showed that the final independent prognostic factors were *LAG3, GZMK,* radiotherapy, age, T stage, M stage and grade. The results of the multivariate Cox regression analysis are shown in supplementary figure 2, and the nomograms are shown in Figure 6A. The areas under the curve (AUCs) of the time-dependent ROC curves were 0.839, 0.802 and 0.769 for 1-, 3-, and 5-year OS, respectively (Figure 6B). The calibration curves of the 1-, 3-, and 5-year survival rates also showed that the nomogram had good prediction ability (Figure 6C–E).

## Discussion

In our study, we first obtained RNA-seq and clinical data of ccRCC patients from the TCGA database. Moreover, ssGSEA and the ESTIMATE algorithm were used to obtain the corresponding immune microenvironment scores, and through consistency clustering analysis, we obtained the relevant immune clusters. The immune microenvironments of these clusters were significantly different, and there were also significant differences between the immunosuppressive sites of the CTLA4 and HLA gene families. The related hub genes were found after performing WGCNA among different clusters, and the prognostic signature constructed by these hub genes and clinical information has great predictive ability. These biomarkers may be useful for RCC immunotherapy.

In recent years, the drug treatment of ccRCC has changed from the era of cytokines to the era of targeted drugs and even the new era of immunotherapy. Immunotherapy has become a choice for ccRCC treatment. ccRCC is characterized by multidrug resistance and biological heterogeneity. It is not sensitive to traditional radiotherapy and chemotherapy. Because there are a large number of inflammatory cells, such as T cells, natural killer (NK) cells, dendritic cells and macrophages, around RCCs, immunotherapy may be an effective treatment (Noessner et al. 2012). With the expeditious development of high-throughput sequencing technology, there are an increasing number of studies on tumor biomarkers using bioinformatics technology by using the RNA-aeq data from lots of databases (Liao, Wang, et al. 2020; Liao, Xiao, et al. 2020; Liao et al. 2019). Therefore, in this study, we explored immune-related biomarkers of ccRCC.

At present, many clinical and basic experimental studies have shown that a variety of factors constitute a unique tumor microenvironment and promote the immune tolerance of tumor cells. One of the most prominent factors is the expression of a variety of negative costimulatory molecules, such as CTLA-4 (Alme et al. 2016), TIM3 (Li et al. 2016), and LAG3 (Ivanova et al. 2020). Due to the poor clinical effect of CTLA-4 and PD-1 in the treatment of KIRC, many researchers are actively seeking new negative costimulatory molecules combined with blocking therapy strategies. Remarkably, LAG3 is considered another promising target for cancer therapy. In addition to its inhibitory effect on T cell activation, it also has a synergistic inhibitory effect with PD-1 in various diseases, such as viral infection, parasites, hematological tumors and solid tumors. For example, the depletion of bovine T cells is caused by cryptosporidium infection, chronic tuberculosis infectious diseases, viral infectious diseases, ovarian cancer and chronic lymphoma (Boer et al. 2013; Imai et al. 2018; Saleh et al. 2019; Chen et al. 2020; Simonaggio et al. 2020).

Although LAG3 is another potential target of immunotherapy, a study on pan-cancer from the TCGA found that although the CD8 + T cell marker CD8a and LAG-3 are strongly coexpressed in most cancers, there are still three obvious exceptions: HPV + head and neck squamous cell carcinoma, RCC and glioblastoma (Panda et al. 2020). Zelba et al. (2019) isolated tumor-infiltrating lymphocytes and autologous peripheral blood mononuclear cells (PBMCs) from patients with primary RCC. After staining and examining the cells with polychromatic flow cytometry, it was found that blocking PD-1 could lead to the upregulation of LAG-3; moreover, the double blocking of PD-1 and LAG-3 and the dual blocking of PD-1 and TIM-3 resulted in an increase in IFNγ release in vitro. It has also been proven that the double blocking of PD-1 and LAG-3 is a promising checkpoint blocking combination for ccRCC.

Another hub gene that we found to have a significant impact on prognosis was *GZMK.* There are five granzyme genes in humans (*GZMA, GZMB, GZMH, GZMK* and *GZMM*). *GZMK* is an apoptotic serine protease secreted by granules. Studies have shown that GZMK has a significant proinflammatory effect. It can induce the secretion of a variety of inflammatory factors, such as interleukin (IL)-1β, IL-6, and IL-8 and monocyte chemoattractant protein-1 (MCP-1). In addition, *GZMK* activated protease activated receptor 1 and induced cell proliferation (Lee et al. 2015). In a phase I multicenter dose escalation study for advanced solid malignancies, in which 36 RCC patients were included and the safety and therapeutic efficacy of the anti-PD 1 antibody MEDI0680 were evaluated, the degree of tumor invasion of CD8+ T cells and the expression of the GZMK gene were also increased after using MEDI0680 (Naing et al. 2019). Moreover, GZMK may be involved in the pathological mechanism of many kinds of tumors, such as colorectal cancer (Zhang et al. 2018), lung cancer (Weng et al. 2016) and breast cancer (Joeckel et al. 2017). GZMK and LAG3 have been confirmed by some studies that they may be related to the pathophysiology of ccRCC, and the nomogram can accurately predict the prognosis of patients, which has certain clinical value.

Our research also has some shortcomings. First, Our research is mainly based on bioinformatics analysis, all the data are from the open database and not from our own experience; thus, in-depth functional verification is needed. Second, there may be some deviations in the data obtained from public databases. Finally, all the samples examined in this study were from patients living in the United States, most of whom were Caucasian, so our results may not be representative of the global population. Therefore, more well-designed experiments with large sample sizes need to be used to further verify our conclusions.

## Conclusion

In our study, we found two immune treatment-related biomarkers: *LAG3* and *GZMK*. We also found that the prognosis of ccRCC patients could be accurately determined by combining their clinical information and the expression levels of *LAG3* and *GZMK*.

## Supplementary Material

Supplemental Material

## Data Availability

The data generated within the study is shown in this manuscript. Any raw data or analysis would be available from the corresponding author upon request.
