# Supplementary figures and images for: Analyzing and validating the prognostic value and immune microenvironment of clear cell renal cell carcinoma
Source: Anim Cells Syst (Seoul). 2022 Mar 29;26(2):52–61. doi: 10.1080/19768354.2022.2056635 (PMC9037198; doi:10.1080/19768354.2022.2056635)

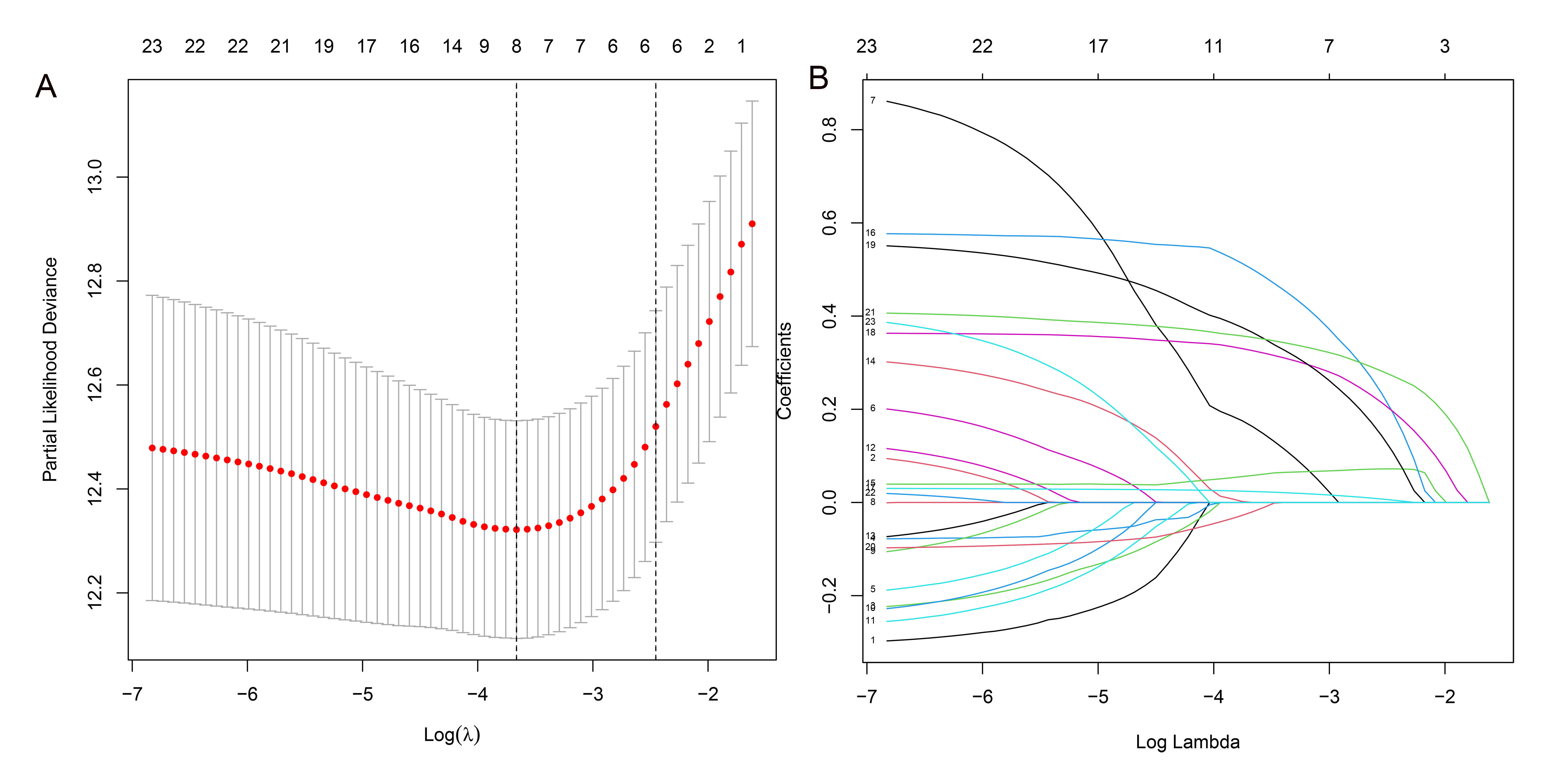

Supplement: Supplemental Material [file TACS_A_2056635_SM4939.zip › S1.jpg]

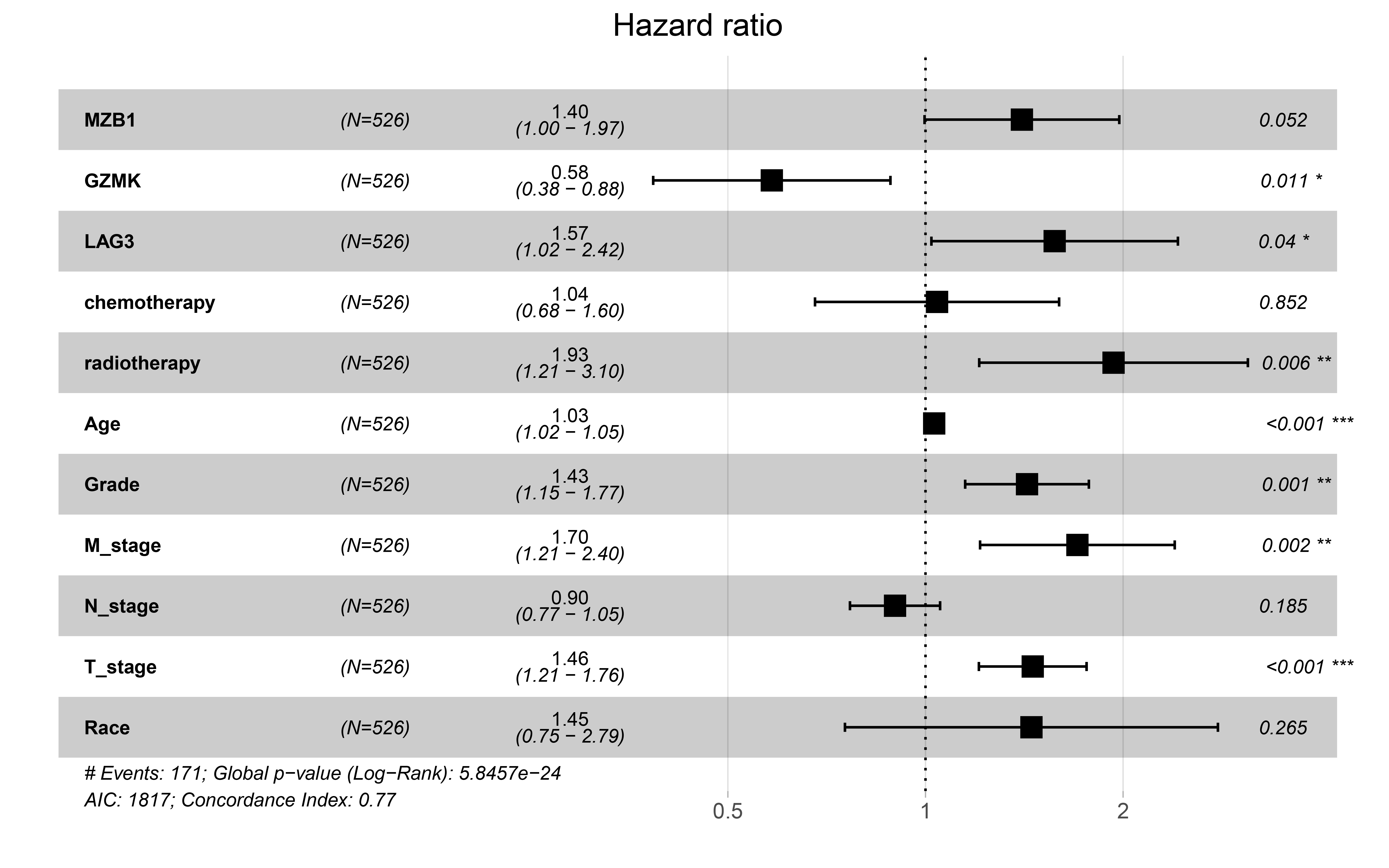

Supplement: Supplemental Material [file TACS_A_2056635_SM4939.zip › S2.jpg]
