# Supplementary material for: Analyzing and validating the prognostic value and immune microenvironment of clear cell renal cell carcinoma
Source: Anim Cells Syst (Seoul). 2022 Mar 29;26(2):52–61. doi: 10.1080/19768354.2022.2056635 (PMC9037198; doi:10.1080/19768354.2022.2056635)
Supplement: Supplemental Material [file TACS_A_2056635_SM4939.zip › Supplementary Data Sheet S1.doc]

#genes with variance in the top 25%

setwd('D:\\kidney\\7wgcna')

options(stringsAsFactors = FALSE);

expro=read.csv('expr.txt',sep = '\t',row.names = 1)

dim(expro)

m.vars=apply(expro,1,var)

expro.upper=expro[which(m.vars>quantile(m.vars, probs = seq(0, 1,

0.25))[4]),]

dim(expro.upper)

write.table(expro.upper,file="geneInput_variancetop0.25.txt",sep='\t',quote=F,row.names=T)

library("WGCNA")

setwd("")	#Set working directory

data=read.table("geneInput_variancetop0.25.txt",sep="\t",header=T,check.names=F,row.names=1)

#Delete normal samples

group=sapply(strsplit(colnames(data),"\\-"),"[",4)

group=sapply(strsplit(group,""),"[",1)

group=gsub("2","1",group)

data=data[,group==0]

datExpr0=t(data)

###Check for missing values

gsg = goodSamplesGenes(datExpr0, verbose = 3)

if (!gsg$allOK)

{

\# Optionally, print the gene and sample names that were removed:

if (sum(!gsg$goodGenes)>0)

printFlush(paste("Removing genes:", paste(names(datExpr0)[!gsg$goodGenes], collapse = ", ")))

if (sum(!gsg$goodSamples)>0)

printFlush(paste("Removing samples:", paste(rownames(datExpr0)[!gsg$goodSamples], collapse = ", ")))

\# Remove the offending genes and samples from the data:

datExpr0 = datExpr0[gsg$goodSamples, gsg$goodGenes]

}

###Sample clustering

sampleTree = hclust(dist(datExpr0), method = "average")

pdf(file = "1_sample_cluster.pdf", width = 17, height = 6)

par(cex = 0.6)

par(mar = c(0,4,2,0))

plot(sampleTree, main = "Sample clustering to detect outliers", sub="", xlab="", cex.lab = 1.5, cex.axis = 1.5, cex.main = 2)

abline(h = 400, col = "red")

dev.off()

clust = cutreeStatic(sampleTree, cutHeight = 400, minSize = 10)

table(clust)

keepSamples = (clust==1)

datExpr0 = datExpr0[keepSamples, ]

traitData = read.table("trait.txt",row.names=1,header=T,comment.char = "",check.names=F)

fpkmSamples = rownames(datExpr0)

traitSamples =rownames(traitData)

sameSample=intersect(fpkmSamples,traitSamples)

datExpr0=datExpr0[sameSample,]

datTraits=traitData[sameSample,]

sampleTree2 = hclust(dist(datExpr0), method = "average")

traitColors = numbers2colors(datTraits, signed = FALSE)

pdf(file="2_sample_heatmap.pdf",width=18,height=6)

plotDendroAndColors(sampleTree2, traitColors,

groupLabels = names(datTraits),

main = "Sample dendrogram and trait heatmap")

dev.off()

enableWGCNAThreads()

powers = c(1:20)

sft = pickSoftThreshold(datExpr0, powerVector = powers, verbose = 5)

pdf(file="3_scale_independence.pdf",width=9,height=9)

par(mfrow = c(1,2))

cex1 = 0.9

plot(sft$fitIndices[,1], -sign(sft$fitIndices[,3])*sft$fitIndices[,2],

xlab="Soft Threshold (power)",ylab="Scale Free Topology Model Fit,signed R^2",type="n",

main = paste("Scale independence"));

text(sft$fitIndices[,1], -sign(sft$fitIndices[,3])*sft$fitIndices[,2],

labels=powers,cex=cex1,col="red");

abline(h=0.9,col="red")

plot(sft$fitIndices[,1], sft$fitIndices[,5],

xlab="Soft Threshold (power)",ylab="Mean Connectivity", type="n",

main = paste("Mean connectivity"))

text(sft$fitIndices[,1], sft$fitIndices[,5], labels=powers, cex=cex1,col="red")

dev.off()

sft

softPower =sft$powerEstimate

adjacency = adjacency(datExpr0, power = softPower)

TOM = TOMsimilarity(adjacency);

dissTOM = 1-TOM

geneTree = hclust(as.dist(dissTOM), method = "average");

pdf(file="4_gene_clustering.pdf",width=12,height=9)

plot(geneTree, xlab="", sub="", main = "Gene clustering on TOM-based dissimilarity",

labels = FALSE, hang = 0.04)

dev.off()

minModuleSize = 50

dynamicMods = cutreeDynamic(dendro = geneTree, distM = dissTOM,

deepSplit = 2, pamRespectsDendro = FALSE,

minClusterSize = minModuleSize);

table(dynamicMods)

dynamicColors = labels2colors(dynamicMods)

table(dynamicColors)

pdf(file="5_Dynamic_Tree.pdf",width=6,height=6)

plotDendroAndColors(geneTree, dynamicColors, "Dynamic Tree Cut",

dendroLabels = FALSE, hang = 0.03,

addGuide = TRUE, guideHang = 0.05,

main = "Gene dendrogram and module colors")

dev.off()

MEList = moduleEigengenes(datExpr0, colors = dynamicColors)

MEs = MEList$eigengenes

MEDiss = 1-cor(MEs);

METree = hclust(as.dist(MEDiss), method = "average")

pdf(file="6_Clustering_module.pdf",width=7,height=6)

plot(METree, main = "Clustering of module eigengenes",

xlab = "", sub = "")

MEDissThres = 0.4#剪切高度可修改

abline(h=MEDissThres, col = "red")

dev.off()

merge = mergeCloseModules(datExpr0, dynamicColors, cutHeight = MEDissThres, verbose = 3)

mergedColors = merge$colors

mergedMEs = merge$newMEs

pdf(file="7_merged_dynamic.pdf", width = 9, height = 6)

plotDendroAndColors(geneTree, cbind(dynamicColors, mergedColors),

c("Dynamic Tree Cut", "Merged dynamic"),

dendroLabels = FALSE, hang = 0.03,

addGuide = TRUE, guideHang = 0.05)

dev.off()

moduleColors = mergedColors

table(moduleColors)

colorOrder = c("grey", standardColors(50))

moduleLabels = match(moduleColors, colorOrder)-1

MEs = mergedMEs

nGenes = ncol(datExpr0)

nSamples = nrow(datExpr0)

moduleTraitCor = cor(MEs, datTraits, use = "p")

moduleTraitPvalue = corPvalueStudent(moduleTraitCor, nSamples)

pdf(file="8_Module_trait.pdf",width=7,height=7)

textMatrix = paste(signif(moduleTraitCor, 2), "\n(",

signif(moduleTraitPvalue, 1), ")", sep = "")

dim(textMatrix) = dim(moduleTraitCor)

par(mar = c(5, 10, 3, 3))

labeledHeatmap(Matrix = moduleTraitCor,

xLabels = names(datTraits),

yLabels = names(MEs),

ySymbols = names(MEs),

colorLabels = FALSE,

colors = blueWhiteRed(50),

textMatrix = textMatrix,

setStdMargins = FALSE,

cex.text = 1,

zlim = c(-1,1),

main = paste("Module-trait relationships"))

dev.off()

###Calculate MM and GS values

modNames = substring(names(MEs), 3)

geneModuleMembership = as.data.frame(cor(datExpr0, MEs, use = "p"))

MMPvalue = as.data.frame(corPvalueStudent(as.matrix(geneModuleMembership), nSamples))

names(geneModuleMembership) = paste("MM", modNames, sep="")

names(MMPvalue) = paste("p.MM", modNames, sep="")

traitNames=names(datTraits)

geneTraitSignificance = as.data.frame(cor(datExpr0, datTraits, use = "p"))

GSPvalue = as.data.frame(corPvalueStudent(as.matrix(geneTraitSignificance), nSamples))

names(geneTraitSignificance) = paste("GS.", traitNames, sep="")

names(GSPvalue) = paste("p.GS.", traitNames, sep="")

for (trait in traitNames){

traitColumn=match(trait,traitNames)

for (module in modNames){

column = match(module, modNames)

moduleGenes = moduleColors==module

if (nrow(geneModuleMembership[moduleGenes,]) > 1){

outPdf=paste("9_", trait, "_", module,".pdf",sep="")

pdf(file=outPdf,width=7,height=7)

par(mfrow = c(1,1))

verboseScatterplot(abs(geneModuleMembership[moduleGenes, column]),

abs(geneTraitSignificance[moduleGenes, traitColumn]),

xlab = paste("Module Membership in", module, "module"),

ylab = paste("Gene significance for ",trait),

main = paste("Module membership vs. gene significance\n"),

cex.main = 1.2, cex.lab = 1.2, cex.axis = 1.2, col = module)

abline(v=0.8,h=0.5,col="red")

dev.off()

}

}

}

probes = colnames(datExpr0)

geneInfo0 = data.frame(probes= probes,

moduleColor = moduleColors)

for (Tra in 1:ncol(geneTraitSignificance))

{

oldNames = names(geneInfo0)

geneInfo0 = data.frame(geneInfo0, geneTraitSignificance[,Tra],

GSPvalue[, Tra])

names(geneInfo0) = c(oldNames,names(geneTraitSignificance)[Tra],

names(GSPvalue)[Tra])

}

for (mod in 1:ncol(geneModuleMembership))

{

oldNames = names(geneInfo0)

geneInfo0 = data.frame(geneInfo0, geneModuleMembership[,mod],

MMPvalue[, mod])

names(geneInfo0) = c(oldNames,names(geneModuleMembership)[mod],

names(MMPvalue)[mod])

}

geneOrder =order(geneInfo0$moduleColor)

geneInfo = geneInfo0[geneOrder, ]

write.table(geneInfo, file = "GS_MM.xls",sep="\t",row.names=F)

#ssGSEA

setwd("D:\\kidney\\1ssgsea")

inputFile="symbol.txt"

gmtFile="immune.gmt"

library(GSVA)

library(limma)

library(GSEABase)

rt=read.table(inputFile,sep="\t",header=T,check.names=F)

rt=as.matrix(rt)

rownames(rt)=rt[,1]

exp=rt[,2:ncol(rt)]

dimnames=list(rownames(exp),colnames(exp))

mat=matrix(as.numeric(as.matrix(exp)),nrow=nrow(exp),dimnames=dimnames)

mat=avereps(mat)

mat=mat[rowMeans(mat)>0,]

geneSet=getGmt(gmtFile,

geneIdType=SymbolIdentifier())

ssgseaScore=gsva(mat, geneSet, method='ssgsea', kcdf='Gaussian', abs.ranking=TRUE)

normalize=function(x){

return((x-min(x))/(max(x)-min(x)))}

ssgseaOut=normalize(ssgseaScore)

ssgseaOut=rbind(id=colnames(ssgseaOut),ssgseaOut)

write.table(ssgseaOut,file="ssgseaOut.txt",sep="\t",quote=F,col.names=F)

#LASSO

library("glmnet")

library("survival")

setwd("D:\\kidney\\8cox\\08.loopTrainTest")

rt=read.table("expTime.txt",header=T,sep="\t",row.names=1,check.names=F) rt$futime[rt$futime<=0]=1

x=as.matrix(rt[,c(3:ncol(rt))])

y=data.matrix(Surv(rt$futime,rt$fustat))

fit <- glmnet(x, y, family = "cox", maxit = 1000)

pdf("lambda.pdf")

plot(fit, xvar = "lambda", label = TRUE)

dev.off()

cvfit <- cv.glmnet(x, y, family="cox", maxit = 1000)

pdf("cvfit.pdf")

plot(cvfit)

abline(v=log(c(cvfit$lambda.min,cvfit$lambda.1se)),lty="dashed")

dev.off()

coef <- coef(fit, s = cvfit$lambda.min)

index <- which(coef != 0)

actCoef <- coef[index]

lassoGene=row.names(coef)[index]

lassoGene=c("futime","fustat",lassoGene)

lassoSigExp=rt[,lassoGene]

lassoSigExp=cbind(id=row.names(lassoSigExp),lassoSigExp)

write.table(lassoSigExp,file="lassoSigExp.txt",sep="\t",row.names=F,quote=F)

#Multivariate Cox regression analysis

library(survival)

library(survminer)

setwd("D:\\kidney\\8cox\\mouticox")

rt=read.table("lassoSigExp.txt",header=T,sep="\t",check.names=F,row.names=1)

rt[,"futime"]=rt[,"futime"]/365

multiCox=coxph(Surv(futime, fustat) ~ ., data = rt)

#multiCox=step(multiCox,direction = "both")

multiCoxSum=summary(multiCox)

outTab=data.frame()

outTab=cbind(

coef=multiCoxSum$coefficients[,"coef"],

HR=multiCoxSum$conf.int[,"exp(coef)"],

HR.95L=multiCoxSum$conf.int[,"lower .95"],

HR.95H=multiCoxSum$conf.int[,"upper .95"],

pvalue=multiCoxSum$coefficients[,"Pr(>|z|)"])

outTab=cbind(id=row.names(outTab),outTab)

write.table(outTab,file="multiCox.xls",sep="\t",row.names=F,quote=F)

pdf(file="forest.pdf",

width = 8,

height = 5,

)

ggforest(multiCox,

main = "Hazard ratio",

cpositions = c(0.02,0.22, 0.4),

fontsize = 0.7,

refLabel = "reference",

noDigits = 2)

dev.off()

riskScore=predict(multiCox,type="risk",newdata=rt)

coxGene=rownames(multiCoxSum$coefficients)

coxGene=gsub("`","",coxGene)

outCol=c("futime","fustat",coxGene)

risk=as.vector(ifelse(riskScore>median(riskScore),"high","low"))

write.table(cbind(id=rownames(cbind(rt[,outCol],riskScore,risk)),cbind(rt[,outCol],riskScore,risk)),

file="risk.txt",

sep="\t",

quote=F,

row.names=F)

library(rms)

setwd("D:\\kidney\\8cox\\mouticox")

rt=read.table("NOM.txt",sep="\t",header=T,row.names=1,check.names=F)

dd <- datadist(rt)

options(datadist="dd")

f <- cph(Surv(futime, fustat) ~ LAG3 + GZMK + Radiotherapy + Age + Grade + M_stage + T_stage, x=T, y=T, surv=T, data=rt, time.inc=1)

surv <- Survival(f)

nom <- nomogram(f, fun=list(function(x) surv(1, x), function(x) surv(3, x), function(x) surv(5, x)),

lp=F, funlabel=c("1-year survival", "3-year survival", "5-year survival"),

maxscale=100,

fun.at=c(0.9, 0.8, 0.7, 0.6, 0.5, 0.4, 0.3,0.2,0.1))

pdf(file="nomogram.pdf",height=6,width=10)

plot(nom,col.grid=c("red","purple","green"))

plot(nom)

dev.off()

setwd("D:\\kidney\\8cox\\mouticox")

library(rms)

library(foreign)

library(survival)

seer<-read.table("NOM.txt",header=T,sep="\t")

ddist <- datadist(seer)

options(datadist='ddist')

units(seer$survival_time) <- "Year"

fcox <- cph(Surv(survival_time,status) ~ LAG3 + GZMK + Radiotherapy + Age + Grade + M_stage + T_stage,surv=T,x=T, y=T,data=seer)

fcox1 <- cph(Surv(survival_time,status) ~ LAG3 + GZMK + Radiotherapy + Age + Grade + M_stage + T_stage,surv=T,x=T, y=T,time.inc = 1,data=seer)

cal1 <- calibrate(fcox1, cmethod="KM", method="boot", u=1, m=150, B=100)

plot(cal1)

fcox3 <- cph(Surv(survival_time,status) ~ LAG3 + GZMK + Radiotherapy + Age + Grade + M_stage + T_stage,surv=T,x=T, y=T,time.inc = 3,data=seer)

cal3 <- calibrate(fcox3, cmethod="KM", method="boot", u=3, m=200, B=100)

plot(cal3)

fcox5 <- cph(Surv(survival_time,status) ~ LAG3 + GZMK + Radiotherapy + Age + Grade + M_stage + T_stage,surv=T,x=T, y=T,time.inc = 5,data=seer)

cal5 <- calibrate(fcox5, cmethod="KM", method="boot", u=5, m=150, B=100)

plot(cal5)
